# Evaluating blinatumomab implementation in low- and middle-income countries: a study protocol

**DOI:** 10.1186/s43058-022-00310-5

**Published:** 2022-06-11

**Authors:** Caitlyn Duffy, Victor Santana, Hiroto Inaba, Sima Jeha, Jennifer Pauley, Liz Sniderman, Niharendu Ghara, Naureen Mushtaq, Gaurav Narula, Nickhill Bhakta, Carlos Rodriguez-Galindo, Heather Brandt

**Affiliations:** 1 Department of Oncology, St. Jude Children’s Research Hospital, 262 Danny Thomas Place, Memphis, TN 38105 USA; 2 Department of Global Pediatric Medicine, St. Jude Children’s Research Hospital, 262 Danny Thomas Place, Memphis, TN 38105 USA; 3 Department of Pediatric Hematology and Oncology, Tata Medical Center, Kolkata, India; 4 Department of Oncology, Aga Khan University Hospital, Karachi, Pakistan; 5 Department of Medical Oncology, Tata Memorial Hospital, Homi Bhabha National Institute, Mumbai, India; 6 Department of Epidemiology and Cancer Control, St. Jude Children’s Research Hospital, 262 Danny Thomas Place, Memphis, TN 38105 USA

**Keywords:** Acute lymphoblastic leukemia, Implementation science, Novel therapies, Low- and middle-income countries, Oncology, Consolidated Framework for Implementation Research, Resource-poor settings

## Abstract

**Background:**

The recent implementation of novel therapies has accelerated progress in pediatric cancer care. Despite the significantly poorer survival of patients in low- and middle-income countries (LMICs), administation complexities and other significant resource barriers have limited the translation of these novel therapies in these regions. This study aims to develop a model that can be used to support the implementation of novel therapies, such as blinatumomab (bispecific antibody therapy for B-cell acute lymphoblastic leukemia [B-ALL]) in LMIC centers, with the long-term goal of developing an implementation framework for similar future efforts.

**Methods:**

In this study, mixed methods will be applied to understand the key contextual considerations that can be accounted for through a training program and prospectively designed implementation activities. The Consolidated Framework for Implementation Research will guide the activities related to implementation evaluation in parallel with a drug donation program. A multidisciplinary research team comprising high- and low-middle income healthcare professionals, industry, and implementation scientists has been assembled with the common goal of improving safe access to blinatumomab. To assess the factors affecting blinatumomab administration, semi-structured interviews with diverse collaborators and quantitative assessments of organizational characteristics will be conducted, together with quantitative and qualitative assessments of feasibility, acceptability, appropriateness, and cost of blinatumomab implementation. A quantitative assessment of stakeholder perceptions of different implementation strategies used as part of the multifaceted approach will also be performed. Finally, we will examine the key domains and processes used and construct the implementation roadmap for translation of novel therapies.

**Discussion:**

This study will rigorously develop an implementation roadmap for translation of novel therapies in low-resource settings. The knowledge gained in the formative assessment will reveal the priority areas and key implementation strategies. Thereby, the resultant roadmap will facilitate future scale-out strategies for novel therapies in LMICs, thus increasing access, building capacity for management, and ultimately improving the care for children in LMICs.

## Contributions to the literature

- This study addresses the large survival gap between children with B-cell acute lymphoblastic leukemia (B-ALL) living in high- vs low- and middle-income countries (LMICs) by designing an approach to facilitate the translation of a novel therapy to children in LMICs.
- Scale-out and sustainability of this initiative require a systematic and scientifically rigorous understanding of the determinants and successful strategies contributing to the effectiveness of resource-adapted and resource-appropriate administration of novel therapies.
- This study demonstrates the value of collaborator engagement from academic, clinical, and industry partners to go beyond traditional humanitarian efforts and improve care delivery for children with B-ALL in LMICs.

## Background

### Novel therapies in pediatric leukemia

Acute lymphoblastic leukemia (ALL) is the most common type of pediatric cancer and is highly curable with chemotherapy and supportive care. In high-income countries (HICs) like the USA, survival for pediatric ALL is > 90% [1]. While most children with B-ALL survive their disease, outcomes for the 15% with relapsed/refractory disease are historically dismal [2]. Scientific advancements and improved understanding of the biology of ALL have led to the development of novel targeted therapeutic approaches. Blinatumomab (Blincyto®) is a member of the bispecific T-cell engager (BiTE) class and has significantly improved survival for these patients [3]. Blinatumomab was first approved by the US Food and Drug Administration (FDA) as a treatment for relapsed/refractory B-ALL in 2014, then for minimal residual disease (MRD)-positive B-ALL in 2018, and is now increasingly incorporated into frontline ALL treatment. Randomized clinical trials comparing the efficacy of blinatumomab to conventional intensive salvage chemotherapy in pediatric relapsed/refractory B-ALL not only show the improved rate of complete remission, overall survival, and disease-free survival with blinatumomab but also lower rates of adverse events (AEs) [4, 5]. As a result of these novel therapies, the survival rate for pediatric cancers in HICs continues to increase.

### Pediatric cancer survival gap

The global burden of childhood cancer disproportionately affects children living in low- and middle-income countries (LMICs) where more than 80% of children with cancer live [6]. Pediatric ALL survival in LMICs is poor and highly variable by region, from 22% in Africa, 52% in Asia, and 61% in Latin America [1]. This survival gap represents one of the largest disparities in global health; addressing this disparity will require multidisciplinary collaboration at a global level [7].

Poor survival in LMICs is multifactorial and linked to the wide range of resource diversity. In low-income countries, limited supportive care, delayed diagnosis, treatment-related mortality, infection, and abandonment are major barriers to care [8]. However, 75% of the world’s population lives in middle-income countries (MICs) [9], and many have strong healthcare infrastructures, supportive care services, risk stratification with molecular diagnostics, and treat ALL patients with adapted protocols based on published studies from HICs. Despite this, survival outcomes in MICs do not match those in HICs. For example, despite the growth in health system capacity in 2005–2014, outcomes for pediatric ALL in Russia, Mexico, Argentina, Brazil, and Belarus have stalled 10–30% below that of HICs [10]. As reported by international oncology providers in upper-middle-income countries, the lack of access to novel therapies is the main barrier to oncology care in these settings [11] and remains a significant hindrance to incremental improvement that has defined pediatric ALL treatment in HICs over the last 60 years. This highlights a significant problem wherein there is no established, evidence-based method to match the supply and demand of effective novel therapies and translate them into real-world settings such that it operationalizes readiness and optimizes patient safety.

### Translation of novel therapies

Medication cost and skepticism related to the cost-effectiveness of novel therapies are often considered the biggest barriers to translation into LMICs [12]. While blinatumomab is costly, translation has the potential to not only improve survival for the most common type of pediatric cancer but also reduce the need for more toxic, resource-intensive therapies in LMICs. Historic drug donation programs modeled after Glivec International Patient Assistance Program (GIPAP) have addressed medication access limited by cost and logistics; however, their potential impact has been curtailed by inadequately accounting for organizational barriers or medication complexity [13, 14]. Blinatumomab, as with other immunotherapies, is a challenge to administration due to idiosyncrasies that extend beyond traditional pharmaceutical barriers (Table 1) [15–18].

**Table 1:** Challenges related to blinatumomab administration and management as well as additional challenges specific to low- and middle-income context

Administration challenges	• Prolonged continuous infusion (1 cycle = 24 h per day for 28 days, typically 2 cycles per patient)• Dedicated intravenous (IV) line• Traditional routine nursing care, such as flushing the infusion line, may push the medicine through the line too quickly and cause an adverse event or overdose• Requires specific types of IV bags, tubing, and ambulatory pump
Management challenges	• Medication initiation requires premedication with steroids and hospital admission (3–8 days) due to the risk of SIRS response. After discharge, patients remain close to care facilities for frequent monitoring in outpatient infusion centers and are often supported by home health services • Interruption in infusion for ≥ 4 h requires readmission to the hospital to restart the infusion • Serious adverse events: cytokine release syndrome, seizure, and other neurotoxicities ○ May result in rapid blood pressure changes, fever, and oxygen requirement ○ May require management in the intensive care unit • Management of adverse events with steroids or expensive medications such as tocilizumab
Examples of additional challenges in LMICs	• Logistical challenges due to medication importation and storage • Limited resources for supportive care (outpatient infusion management, home health capabilities, inpatient bed availability, monitoring labs, ambulatory pumps, IV bags and tubing, medications to manage adverse events) • Transportation challenges impeding rapid return to the hospital setting

*LMICs* Low- and middle-income countries, *IV* Intravenous, *SIRS* Systemic inflammatory response syndrome

Lessons from the treatment of HIV have demonstrated that even complex and expensive treatments are possible when accompanied by innovative treatment models and new investments [12]. Therefore, facilitating the successful adoption of medications like blinatumomab in LMICs requires an approach that addresses cost but also an implementation approach to maximize existing resources and address therapeutic challenges and barriers related to organizational context [19]. Currently, there is no implementation framework for novel pediatric cancer interventions in a global setting. Therefore, we propose INOVATING (Implementing NOVel Agents & Translating Innovation Globally), a non-therapeutic project to facilitate the implementation of novel agents in resource-limited countries to parallel the Blincyto® Humanitarian Access Program (BHAP), a newly established drug donation program.

### Pre-implementation

In December 2019, St. Jude Children’s Research Hospital (St. Jude) formed an academic-based partnership with the blinatumomab manufacturer, AMGEN, and created BHAP. BHAP is not a research endeavor, but akin to GIPAP, developed as a drug-only donation program on a per-patient basis to improve access to blinatumomab for patients in LMICs where it was not commercially available. All components of patient indications, management, and supportive care follow the FDA approval information, supplemented by peer-reviewed publications. These components are further resource-adapted to ensure patient safety and maximal benefit to patients.

**Fig. 1:**
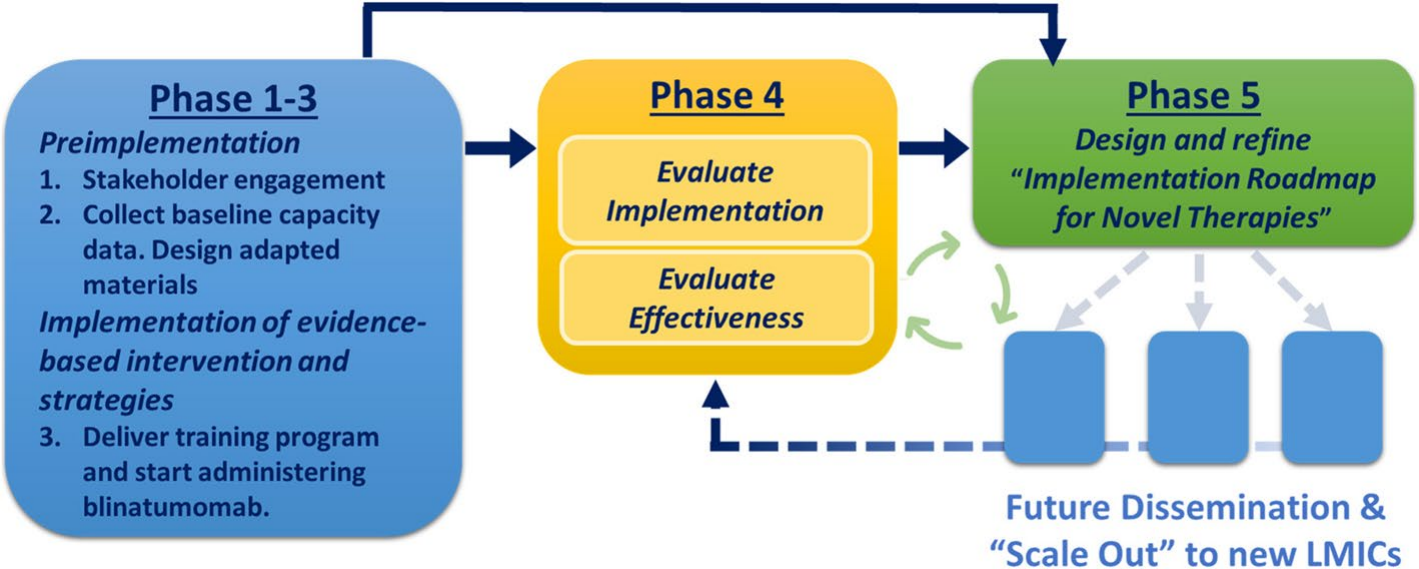
INOVATING 5 phase program plan. The INOVATING program takes place across 5 phases. To date, phases 1–3 are complete, and this proposed protocol outlines the efforts in the evaluation of implementation and effectiveness at LMIC sites. The goal of phase 4 is to create an informed implementation roadmap for novel therapies as phase 5 that can be used to scale out this program to future LMIC sites. LMICs, low- and middle-income countries

The INOVATING program is planned over five phases (Fig. 1).

Relevant constructs from the Consolidated Framework for Implementation Research (CFIR) were used in the planning and execution and will guide the evaluation phase of this approach [20]. CFIR was selected because it is a determinant-type framework focused on identifying contextual factors that influence the effectiveness and facilitate or hinder implementation, and it has been well operationalized in both high-income and low-middle income settings [21, 22]. This program will use implementation research methodology to understand the impact of determinants and specific strategies on blinatumomab adoption and iteratively integrate knowledge to refine the program and create a resource-informed, reproducible, scalable, and adaptable model, the “Implementation Roadmap for Translation of Novel Therapies,” hereafter referred to as the Implementation Roadmap.

### Phase 1: Stakeholder engagement

Stakeholder engagement has been identified as a strategy to increase the relevance and impact of evidence-based interventions [23]. As the first step in this program, a diverse group of collaborators was assembled to develop a comprehensive understanding of multilevel barriers and facilitators impacting blinatumomab implementation in LMICs. Collaborators represent three main groups: (1) implementation scientists and multidisciplinary pediatric oncology providers from St. Jude with experience in administering blinatumomab and in global health, (2) industry representatives from AMGEN Access to Medicine, and (3) multidisciplinary pediatric oncology providers from LMIC sites, with additional partnerships with a nongovernmental organization (NGO) (e.g., Direct Relief) to facilitate logistics of drug importation and delivery.

Three LMIC centers served as pilot sites for the BHAP program and informed the protocol development. These large, pediatric cancer referral hospitals are located within a common geographic region in India and Pakistan and were selected to tailor materials to specific regional and cultural needs, facilitate a support network between neighboring centers, and promote the growth of regional capacity. Prior to the founding of the BHAP program, these sites had expressed interest in administering blinatumomab by submitting requests to the pharmaceutical company.

### Phase 2: Baseline data collection and program planning

The BHAP Core Team, comprised of multidisciplinary representatives from St. Jude and AMGEN, reviewed and assimilated the institutional administration guidelines, prescribing information, and relevant literature from HICs institutions [15–18] and identified the key administration and management issues for blinatumomab. This helped identify the core domains (indication, dose, and duration) of blinatumomab delivery. The BHAP core team designed a questionnaire to characterize the baseline capacity at LMIC sites, including hospital funding, clinical infrastructure, intensive care ward capacity, nursing care, eligible patient population, standardized operating procedures, medication procurement procedures, advanced treatment experience, and training needs for physicians, nurses, and pharmacists. The 64-question survey was reviewed for content, cultural variations, and ease of use by multidisciplinary international experts. One local physician at three LMIC pilot centers completed the survey, and responses were supplemented through informal discussion with the BHAP core team. The modifiable elements from these preliminary investigations traced back to two CFIR domains, the inner setting (e.g., implementation climate, culture, readiness) and the intervention characteristics (e.g., complexity, adaptability, cost, relative advantage), which are consistent with the findings from a systematic review by Means et al. demonstrating inner setting and intervention characteristics were the most utilized CFIR domains in LMIC settings [22]. Based on BHAP Core Team consensus, recommended best practices for blinatumomab administration were preemptively adapted to include reduced frequency laboratory monitoring, inpatient-only administration, AE management, and standardized bag change schedule. With complex interventions, we expect additional barriers to emerge during implementation and will assess the needs for further adaption based on experience with pilot sites.

### Phase 3: Training program design and delivery

In order to prioritize patient safety and to build knowledge base and skills for the medical team to recognize and respond to AEs, we selected a training program as our primary strategy. The BHAP core team created learning objectives informed by LMIC preliminary capacity assessments and site-specific multidisciplinary training needs to tailor the program to the supportive care capacity of sites. These objectives guided the content creation for a comprehensive, context-adapted training program, including didactic presentations, treatment templates, administration and management guidelines, role-specific workflow materials, and caregiver handouts. Materials were developed by the St. Jude multidisciplinary team, and the content was reviewed by the BHAP core team. Given the COVID-19 pandemic restrictions, we transitioned to an online program, with virtual synchronous sessions and recordings posted to an online classroom on the Cure4Kids platform (http://www.cure4kids.org) along with workflow materials. For pilot sites, all training materials and surveys were administered in English; however, translation and validation will be performed based on the needs of future sites. Training was delivered to 78 healthcare providers (25 physicians, 36 nurses, and 17 pharmacists) and sessions conducted independently for each pilot site as two 90-min live, interactive virtual sessions. The content for the first session focused on general information related to blinatumomab indications, mechanism of action, efficacy, and AE profile and was delivered to the combined multidisciplinary team. The second session were provider-specific, with content focused on the management issues related to roles. These didactic sessions were facilitated by a St. Jude pediatric oncologist, a nurse, and a pharmacist, with time for group discussion. The training meetings highlighted the importance of prompt response when managing patients on immunotherapy and modeled risk-reducing communication techniques between providers. After the multidisciplinary team attended the two training sessions, centers could apply for blinatumomab on a per-patient basis, at which point the medication would be shipped and sites could begin administration.

### Study aims and research questions

The overall goal of the next stage, phase 4 (Fig. 1), of the INOVATING program is to formally evaluate the key determinants of novel therapy administration in a resource-limited setting and then develop a generalizable “Implementation Roadmap” to reduce disparities in time to translation and facilitate less costly implementation of effective, newer treatments with lower toxicity profiles.

The aims of this study are to (1) conduct a formative evaluation to assess the determinants that influence the implementation of blinatumomab in LMIC settings; (2) evaluate how different components of the multifaceted strategy package influence implementation, provider knowledge, competence, clinical effectiveness, and safety of drug preparation, administration, and management; and (3) use data collected to inform an “Implementation Roadmap” to speed the translation of blinatumomab in other lower resource settings.

This study seeks to answer the following research questions: (1) Can blinatumomab be adapted for LMIC settings, and is it feasible, acceptable, and appropriate? (2) How does an adapted multidisciplinary training program affect these implementation outcomes? (3) Are there contextual factors (readiness, capacity, perceived complexity) that correlate with successful implementation? (4) What key strategies facilitate implementation and are they similarly prioritized by different stakeholder groups? (5) What is stakeholder satisfaction with this implementation-guided approach? (6) Is the effectiveness of this adapted administration of blinatumomab similar to that documented in high-income settings?

### Methods

This convergent, mixed methods study addresses stated research questions based on the CFIR framework. Table 2 lists data sources. Qualitative methods include open-ended questions, stakeholder interviews, and meeting notes. Quantitative methods include surveys and secondary clinical and costing data. Methods will define the barriers and facilitators and explore the impact of these barriers and strategies on implementation outcomes, including feasibility, acceptability, appropriateness, and clinical effectiveness of blinatumomab in resource-limited settings using an effectiveness-implementation hybrid type 2 case series design. Due to ethical implications and prioritization of patient safety in these centers, randomization is not possible.

**Table 2:** Study data sources including data collector, data collection method, correlation to specific CFIR domain, and target study participant. Matrix of data collection methods

Type of data	Data collectors	Data collection method	CFIR domain	Study participants
Primary data	BHAP core team	Hospital assessment questionnaire	Inner setting	Pilot center physician champion
	St. Jude research team	Provider knowledge assessment	Inner setting	Multidisciplinary pilot center providers
		Organization Readiness for Implementing Change (ORIC)	Intervention characteristics	Pilot center implementation team
		Implementation Climate Scale (ICS)	Inner setting	Pilot center implementation team
		Complexity of intervention assessment	Intervention characteristics	Pilot center implementation team
		Implementation outcome surveys (Feasibility of Intervention Measure, Acceptability of Intervention Measure, Intervention Appropriateness Measure)	Intervention characteristics	Pilot center implementation team
		Strategy ranking survey	Process	Pilot center implementation team
		Stakeholder interviews (barrier assessment, informed adaptation of patient eligibility criteria and training materials, strategy prioritization, and assessment)	Inner setting Process Intervention characteristics Outer setting	Key stakeholders
		Meeting notes	Process	Key stakeholders
		Group interview (strategy analysis, feedback on training strategy, implementation outcome, team dynamics, identifying unanticipated challenges, and adaptation of implementation, provider satisfaction)	Inner setting Process Intervention characteristics	Pilot center implementation team
Secondary data	Local site providers	Medication interruption record	Intervention characteristics	Blinatumomab recipients
	Local site providers	Adverse event reporting, severity, and number of events	Intervention characteristics	Blinatumomab recipients
	Local site providers	Disease outcomes (MRD status, remission status, proceed to transplant, mortality)	Intervention characteristics	Blinatumomab recipients
	Local site providers	Participant satisfaction	Intervention characteristics	Patient and family
	Local site providers	Baseline patient characteristics	Intervention characteristics Inner setting	Blinatumomab recipients
	Local site providers	Delivery cost of blinatumomab	Intervention characteristic	Blinatumomab recipients
Process indicators	St. Jude research team	Attendance of training sessions, utilization of web-based resources, and proportion of patients enrolled in the BHAP program	Process	Pilot center providers

*BHAP* Blincyto® Humanitarian Access Program, *CFIR* Consolidated Framework for Implementation Research, *MRD* Minimal residual disease

The results will yield an in-depth understanding of key determinants and prioritization of key implementation strategies, which will be assembled into a framework to facilitate real-world scalability of blinatumomab in future centers.

### Collaborators and setting

This study is being conducted in collaboration with BHAP. LMIC sites where blinatumomab is not commercially available were identified and stratified into two groups: group 1, characterized by established pediatric cancer units, experienced bone marrow transplant centers, and specialized chemotherapy pharmacists; group 2, characterized as developing pediatric cancer and bone marrow transplant programs (autologous only or none) and overlapping nursing and pharmacist responsibilities.

The experience in the three group 1 pilot sites will inform the characterization of the future grouping in the model to facilitate expansion into future sites.

### Study participants

The training program will be administered to all multidisciplinary pediatric oncology staff at each site. Evaluation data will be collected from multiple stakeholders: LMIC pediatric oncology staff, pilot site implementation team, pharmaceutical industry representatives, and multidisciplinary academic providers at St. Jude. The study will also collect secondary data from ~ 50 children enrolled in the BHAP program who received blinatumomab. Table 3 describes the inclusion criteria.

**Table 3:** Inclusion criteria for primary and secondary data collection

**Primary data study participant group**	**Primary data inclusion criteria**
Local site physician champion	Submit an application for BHAP program enrollment
Multidisciplinary local site pediatric oncology staff	Attend both 90-min synchronous training sessions or watch session recording online
Local site implementation team	Will include a minimum of 2 physicians, 2 nurses, and 1 pharmacist at each LMIC site, who attended both training sessions and participated in frontline management of patients receiving blinatumomab in the inpatient setting
Key stakeholders	Representatives from the pharmaceutical company actively involved in the drug donation program Representatives from St. Jude actively involved in the BHAP program LMIC site implementation team
**Secondary data study participant group**	**Secondary data inclusion criteria**
Patients	Satisfy BHAP program enrollment criteria to receive blinatumomab, parental consent to receive blinatumomab (obtained by a local physician), and initiate blinatumomab infusion

*LMIC* Low- and middle-income countries, *BHAP* Blincyto® Humanitarian Access Program

### Study recruitment and informed consent

This protocol was approved by the St. Jude Institutional Review Board and will be reviewed by LMIC local participating sites’ ethics committees. Recruitment will occur after the completion of the baseline BHAP administrative application and the hospital assessment questionnaire for the drug donation program. Participation in the BHAP program does not necessitate participation in this implementation research study, and AMGEN will not be involved in recruiting participants for the implementation study. If sites agree to participate, the baseline information collected for the BHAP program enrollment will be included in the study.

At LMIC sites, all pediatric oncology staff who participate in the training program will complete a brief knowledge assessment survey. However, the remainder of the implementation evaluation will occur through a site-specific implementation team. This team will be identified by the site PI and consists of multidisciplinary members who have demonstrated organizational leadership in the implementation of blinatumomab and will contain at least two physicians, two nurses, and one pharmacist. This group selection will satisfy pragmatic constraints due to limited resources and time in LMIC settings but will also provide rich representation within and between different provider roles within an institution and thematic saturation across multidisciplinary experts. These providers will give consent to complete additional surveys and participate in semi-structured interviews.

Secondary data related to implementation costs and routine clinical care will be collected. Deidentified patient information will be required to confirm patient suitability for participation in the BHAP program and for routine monitoring during medication administration and clinical care.

### Aim 1: To conduct a formal assessment to characterize determinants that influence blinatumomab implementation in LMICs

For aim 1, this study will use mixed methods to evaluate the hypothesis that barriers related to inner setting and intervention characteristics impact the implementation of blinatumomab in LMICs. This will include the assessment of (1) capacity, (2) key barriers (readiness, climate, intervention complexity, cost), and (3) perceptions of leading indicator implementation outcomes (feasibility, acceptability, and appropriateness) from multiple stakeholders. For the three pilot sites, implementation assessments will occur 1 year after the training; however, for future sites, these will be part of the upfront baseline assessment. Assessments will be repeated at 3, 6, and 12 months post-training to characterize changes in perceived barriers and implementation outcomes over time (Table 4).

**Table 4:** Assessment administration time points, target respondents, and frequency. Measurement tools including the type of data, key respondents, frequency, and time points of administration

Survey topic	Method	Respondent(s)	Frequency	Time point(s)
**Hospital assessment questionnaire**	QUAN	LMIC site physician champion	1 time	Prior to BHAP enrollment
**Complexity assessment**	QUAN	Implementation team	4 times	Pre-training, 3 and 6 months, and 1-year post-training
**Implementation Climate Scale (ICS)**	QUAN			
**Organization Readiness for Implementing Change (ORIC)**	QUAN			
**Acceptability of intervention (AIM)**	QUAN			
**Intervention Appropriateness Measures (IAM)**	QUAN			
**Feasibility of Intervention (FIM)**	QUAN			
**Knowledge assessment: nurses**	QUAN	All nurses	4 times	Pre-training, 3 and 6 months, and 1 year post-training
**Knowledge assessment: pharmacy**	QUAN	All pharmacists		
**Knowledge assessment: physician**	QUAN	All physicians		
**Training material feedback survey**	QUAL	Implementation team	1 time	3 months post-training (after the 1st patient received blinatumomab)
**Semi-structured group interviews:** **1) Training program feedback and site-specific barriers** **2) Site-specific fidelity vs adaptation**	QUAL	Implementation team	2 times	3 months post-training (after the 1st patient received blinatumomab) and 12 months post-training
**Implementation strategy feedback**	QUAN	Key stakeholders (implementation team, industry, St. Jude)	1 time	Annual review
**Program evaluation group interview**	QUAL			
			1 time	Annual review

*LMICs* Low- and middle-income countries, *BHAP* Blincyto® Humanitarian Access Program, *QUAN* Quantitative, *QUAL* Qualitative

We selected well-established measurement tools that have been validated in HICs and were chosen because they are pragmatic, reviewed and agreed upon by stakeholders, and when compiled have less than 50 questions that can be completed in less than 30 min. The quantitative surveys to assess barriers include the Implementation Climate Scale (ICS) [24], Organizational Readiness for Implementing Change (ORIC) [25], and a complexity assessment. Implementation outcome survey assessments include Acceptability of Intervention Measure (AIM), Intervention Appropriateness Measure (IAM), and Feasibility of Intervention Measure (FIM) [26]. These measures of implementation outcomes were selected for their salience with stakeholders and because they are considered “leading indicators” of implementation success for early-stage initiatives [26]. Survey data collected online through Qualtrics will be housed at the Department of Global Pediatric Medicine at St. Jude.

The research team will conduct two semi-structured group interviews with the implementation team to collect additional insights into the site-specific determinants. Interview #1 will be done 3 months after training and after the site has started administering blinatumomab. The CFIR Interview Development Tool will guide the development of the interview guide and will be adapted based on experience with the pilot sites [27]. The team will ask open-ended questions about the implementation processes, barriers, and facilitators and will elicit feedback on the training program. Interview #2 will be done 1 year after training, wherein the research team will ask open-ended questions on site-specific fidelity vs adaptations to recommended administration guidelines, strategies, emerging determinants, costs, team dynamics, adequacy of training program, utility of standardized documentation tools, and parent perception of treatment.

Interviews will be audio-recorded and transcribed verbatim, and the team will use thematic coding methods to analyze and interpret the results. Written transcripts will be reviewed independently by two research team members to identify emergent themes to build a codebook. Then, each transcript will be reviewed again using the codebook. The thematic coding process will focus on process improvement.

Descriptive statistics will be used to characterize the barriers and facilitators to blinatumomab implementation in LMICs. This will include describing core components of implementation, such as site infrastructure, climate, readiness, and intervention complexity. The survey will be summarized by frequency tables and cross-tabulation to study any associations. The chi-squared and Fisher’s exact test will be used to assess the associations. Comparisons will be made with assessments of barriers done over time (pre-training; 3, 6, and 12 months post-training). Univariate and multivariable logistic regression analysis will be used, as appropriate, to study the correlations among scores of clinical infrastructure, readiness, climate, and intervention complexity with implementation outcomes (feasibility, acceptability, and appropriateness) within and across institutions. Collectively, this information will be used to understand barriers and site-specific adaptations to modify the strategy approach and training program for medication delivery.

### Evaluation of implementation cost

The cost of novel therapies is frequently cited as the foremost barrier to implementing therapies in LMICs [28]. As part of the BHAP program, blinatumomab is donated and a partner NGO facilitates distribution. Therefore, this protocol will focus on the cost following drug delivery and will conduct a microcosting analysis related to training, supportive care management, and medication storage. Data will be collected on elements related to drug delivery as an implementation outcome, and observed AE data will be mapped against the data from expected costs from the resource-adapted best administration and management best practices. Prices for all line items will be determined from billing invoices at each hospital or using standardized assumptions. The expected administration costs and observed AE costs will be collated and reported using a previously published framework from prior costing analyses conducted in El Salvador, Ghana, and Mexico in order to ensure complete costs are accounted for [29]. As part of the annual Program Evaluation Group semi-structured interview, open-ended questions will be asked to assess the time and resource costs to determine the investments related to program initiation, delivery, and maintenance and to explore how remaining costs impact equitable distribution.

As an exploratory objective of this study, we will work with BHAP partners to document costs related to the acquisition including drug manufacturing, coordination, and shipping pending data availability as these data are relevant from a sustainability outcome perspective.

### Aim 2: To evaluate a multifaceted implementation strategy package, evaluate the training program, and evaluate how the strategy package influences implementation, clinical effectiveness, and safety of medication preparation, administration, and management

Based on a preliminary understanding of the key determinants and collaborator consensus for program development, a general program outline was designed. Discrete Expert Recommendations for Implementing Change (ERIC) strategies [30] were retrospectively identified, linked to specific CFIR domains [21], and assembled into a multifaceted 5-phase approach which describes the processes utilized to facilitate blinatumomab implementation in LMICs (Table 5). As an implementation effort focused heavily on formative evaluation, we have identified several strategies that target the organizational level used throughout this program [31]. A series of qualitative and quantitative evaluations will be used to assess the different components of the strategy plan to understand stakeholder perception of this approach and determine the effectiveness of the training program and clinical effectiveness.

**Table 5:** Multifaceted strategy by program phase. Compilation of discrete ERIC strategies retrospectively mapped to each phase in the INOVATING program

Pre-implementation		Active implementation		Post-implementation
Phase 1: Establishing partnerships	Phase 2: Collecting baseline data and planning	Phase 3: Implementing evidence-based strategies	Phase 4: Supporting and monitoring implementation	Phase 5: Sustainability and maintenance
1. Obtain formal commitments 2 .Develop resource sharing agreements 3. Use advisory boards and workgroups 4. Work with educational institution 5. Develop academic partnerships 6. Access new funding 7. Conduct local consensus discussion 8. Identify and prepare champions	1. Conduct local needs assessment 2. Assess for readiness and identify barriers and facilitators 3. Organize clinician implementation teams	1. Develop educational materials 2. Conduct educational meetings 3. Distribute educational materials 4. Provide local technical assistance 5. Provide ongoing consultation 6. Develop and implement tools for quality monitoring 7. Remind clinicians	1. Facilitation 2. Use an implementation adviser 3. Purposely re-examine the implementation 4. Promote adaptability 5. Promote network weaving 6. Tailor strategies 7. Capture and share local knowledge 8. Audit and provide feedback 9. Obtain and use patient/consumers and family feedback	1. Develop a formal implementation blueprint 2. Make training dynamic 3. Use train the trainer 4. Stage implementation scale up 5. Use data experts 6. Visit other sites

*ERIC* Expert Recommendation for Implementing Change

### Training program evaluation

The resource-adapted training program will be evaluated in three ways. First, a knowledge assessment survey will be administered to all pediatric oncology providers who participated in the BHAP training program at LMIC sites. For pilot sites, this will be administered as part of a nested post-training assessment. Future sites will receive a baseline survey prior to training and will be repeated at 3, 6, and 12 months post-training to determine the role-specific knowledge related to the preparation, administration, and management of patients receiving blinatumomab. Second, the implementation team will complete an online Training Material Feedback Survey, designed by St. Jude, and participate in semi-structured interview #1 with the site-specific implementation team. The team will ask open-ended questions on the utility of the training program and workflow tools, experience with real-world administration, and additional training needs. Third, a standardized documentation tool was designed a priori by the multidisciplinary providers at St. Jude to record infusion interruptions and will be tested in pilot sites and adapted based on this cohort. It will be completed by the treating physicians and bedside nurses and returned for analysis. This will be an additional way to assess the effectiveness of training, hypothesizing that if training provides sufficient resource-adapted information, prolonged interruptions related to infusion issues will be minimized by standardization.

Comparisons between pre- and post-training knowledge assessment surveys will be made to assess the adequacy of training materials in providing adequate and retained knowledge of drug administration. Comparisons will also be made with assessments done over time (at 3, 6, and 12 months post-training). McNemar’s test will be used to assess the change from pre- vs post-training.

### Strategy assessment

Previous work has characterized the utility of different implementation strategies in HICs [32]; however, we do not know how different collaborator groups involved in LMIC implementation efforts will resemble those conclusions. Collaborator engagement is particularly important for the real-world application of oncology treatments, and sustainability of the drug donation program relies on input, coordination, and alignment from multiple stakeholders. Therefore, a quantitative implementation strategy feedback survey (Table 4) will be administered to assess the feasibility and importance of different implementation strategies utilized in each phase of the program to representatives from each respective stakeholder group (St. Jude, Amgen, and local implementation team) 12 months after training. After completing the survey, the research team will compare and contrast the findings between different stakeholder groups. At the annual Program Evaluation Group interview, the research team will then ask open-ended questions about experience with the different strategies to stakeholders from the three groups. During these meetings, researchers will take field notes and use thematic analysis to interpret the results. The focus of the thematic coding process is process improvement.

### Clinical effectiveness in a real-world setting

To assess the clinical effectiveness of blinatumomab adapted to LMIC contexts, the research team will assess the quality of administration and examine patient outcomes. To assess the quality, a standardized documentation tool was designed to record infusion interruptions and track the frequency and severity of AEs related to blinatumomab with a focus on AEs observed in HIC (as defined by Common Terminology Criteria for Adverse Events [CTCAE] v5.0) [33]. These will be completed by treating physicians and bedside nurses and analyzed at St. Jude. To assess the patient outcomes, the local site physicians will collect deidentified clinical data. These secondary data are available as part of routine clinical care and include patient enrollment criteria, AE frequency and severity, and clinical outcomes including ALL disease status after cycles 1 and 2 of blinatumomab, duration of each cycle, and short- and long-term patient outcomes that include relapse, subsequent management including bone marrow transplant, and mortality at 12 months after administration. This information will serve as a surrogate clinical measure as part of the summative evaluation to determine the adequacy of training programs and identify the domains for implementation program adaptation (e.g., patient suitability criteria, administration method, and supportive care) to improve utilization in current and future sites. Clinical data will be entered into the REDCap database by local physicians who have completed Collaborative Institutional Training Initiative (CITI) training and a Data Abstraction training module on Cure4Kids.

### Process evaluation

We will perform a process evaluation using the Reach, Effectiveness, Adoption, Implementation, and Maintenance (RE-AIM) framework to characterize the reach, adoption, and implementation of the BHAP program [34]. This will include analysis of training participation, user engagement and utilization of online training materials, and breadth of patient selection.

### Aim 3: Prepare an implementation roadmap reflecting important determinants of implementation success and implementation strategies to support the translation of blinatumomab into future LMICs.

In aim 3, the results from analyses for aims 1 and 2 will be compiled to fully characterize the relevant contextual barriers, key implementation strategies, implementation outcomes, and areas of adaptation vs fidelity to facilitate the successful implementation of blinatumomab (Table 6).

**Table 6:** Data use to address specific research questions. Key research questions in the INOVATION program mapped to specific measurement tools used to address each question

Research question	Data source
*(1) Can blinatumomab be adapted for LMIC settings, and is it feasible, acceptable, and appropriate?*	1) Implementation outcomes assessments (FIM, AIM, IAM, cost) 2) Semi-structured interviews 3) Program evaluation group interview
*(2) Does an adapted multidisciplinary training program improve these implementation outcomes?*	1) Knowledge assessments 2) Implementation outcomes assessments (FIM, AIM, IAM, cost)
*(3) Are there contextual factors (readiness, capacity, perceived complexity) that correlate with successful implementation?*	1) Hospital assessment questionnaire 2) Barrier assessments: complexity assessment, ICS, ORIC 3) Implementation outcomes assessments (FIM, AIM, IAM) 4) Semi-structured interviews
*(4) What are the key strategies as perceived by stakeholder groups and are these the same between different groups?*	1) Implementation strategy feedback 2) Program evaluation group interview
*(5) What is stakeholder satisfaction with this implementation-guided approach?*	1) Semi-structured interviews with implementation teams 2) Program evaluation group interview
*(6) Is the effectiveness of this adapted administration of blinatumomab the same as what has been documented in high-income settings?*	1) Patient enrollment information 2) Patient safety record: adverse event reporting 3) Management records: interruption record 4) Clinical outcome data

*LMICs* Low- and middle-income countries, *FIM* Feasibility of Intervention Measure, *AIM* Acceptability of Intervention Measure, *IAM* Intervention Appropriateness Measure, *ICS* Implementation Climate Scale, *ORIC* Organizational Readiness for Implementing Change

The research team will assemble an informed implementation process model, “Implementation Roadmap” that reflects the core domains and modifiable variables based on the unique context, characteristics of the intervention, and stakeholder values. This model will contain “standard elements” generalizable to all centers, which include a phased strategy plan, readiness assessment, training materials, workflow documents, an assessment package to monitor implementation, budget impact models, and tools to support local adaptation processes. It will also include additional variables or strategies that can be included based on the unique needs and resources of the institution. Version 1.0 of this model will be informed from the three pilot institutions and then undergo a review by a focus group and stakeholder qualitative survey and then serve as a mechanism to scale-out access to blinatumomab. With each iteration of implementation at new institutions, new findings from the evaluation process will be incorporated to further refine the model to improve its utility and generalizability to a wider variety of resource settings.

## Discussion

This implementation effort paired with a humanitarian drug donation program is the first of its kind to facilitate the translation of a complex, novel pediatric cancer medication in LMICs and is innovative in its approach to partnership engagement, cost, adaptation, and vision for scalability.

The historical context of novel therapy translation was heavily considered throughout the development of this program. Unfortunately, this has been negatively impacted by experimental approaches, using unsustainable, unadapted, and inappropriate interventions resulting in concerns for patient safety and mistrust of providers and communities. From program inception, it was clear that an effort of this complexity and magnitude would not be feasible through the work of a single group or institution due to the scale of documented barriers such as financial limitations, training needs, and regulatory constraints which necessitated expertise from many different organizations for effective program planning and execution [7]. Additionally, the critical relationship between an ethical approach with a context-informed intervention and ultimate program feasibility, acceptability, and appropriateness necessitated upfront engagement of LMIC collaborators to provide critical contextual input, transparently address barriers, and innovate potential solutions. Partner engagement extended deep within the multidisciplinary healthcare team and wide to include representatives from academic medicine, implementation scientists, LMIC clinicians, and industry to overcome primary barriers related to cost and NGO partners with experience in medication importation and regulatory logistics. To provide ongoing support for implementation within BHAP, clear operating procedures and accountability were established by defining a steering committee charter and with biweekly meetings to review program processes, pilot site progress, and provide technical assistance to LMIC partners. Not only was provider support and engagement prioritized, but it was also essential to address the needs and establish trust with patients and caregivers. Initial discussions with LMIC clinicians identified hesitancy from local families to receive unfamiliar medications. Therefore, as part of the training materials, a handout for caregivers was created by the St. Jude multidisciplinary team based on information from the Together website (https://together.stjude.org/en-us/) and included information on blinatumomab’s established efficacy, mechanism of action, safety issues, side effects, and then resource adapted to prepare families for medication administration. Although the COVID-19 pandemic limited the research team’s direct interaction with families, these materials can be used and translated as needed by local providers to facilitate discussions with families with whom they already have established relationships. As part of semi-structured interview #2, the research team will inquire about caregiver and child perceptions related to medication delivery and refine materials based on this feedback. The importance of family and provider perception has also played an important role in selecting a hybrid study design that both examines implementation outcomes and provides evidence for local effectiveness which we hypothesize will contribute to long-term appropriateness, acceptability, and sustainability for blinatumomab implementation.

The cost associated with novel therapies has been a significant barrier to LMIC translation [28]. For blinatumomab, the wholesale drug costs in the USA are approximately US$127,623.00–237,557.60 (based on cost per vial, range of vials used per cycle, and standard 2-cycle course) and represent the primary driver of overall therapy cost [35]. While cost-effectiveness studies based on simulation have demonstrated that blinatumomab is cost-effective when compared to traditional salvage chemotherapy for adults in HICs [34], this upfront cost remains prohibitive for most patients and health systems globally, especially in LMICs [36]. Through industry partnership in the BHAP program, blinatumomab donation and importation has the potential to drastically reduces these costs, thereby opening the door to broader drug access. However, as a drug-only donation program, the remaining costs attributable to storage, administration, and supportive care are not insignificant, and likely represent unaffordable expenses if passed on to patients in LMICs.

The proposed adaptation of blinatumomab was considered based on improving safe delivery and incorporating cost-reducing measures. All intervention adaptations were made with the goal of decreasing associated costs, except for one related to the treatment location. Typical blinatumomab management in an HIC requires 3–8 days inpatient for medication initiation, followed by discharge to home with close outpatient follow-up for the remainder of the 28-day cycle. However, in LMICs, there are often greater infrastructure barriers, less support for emergency medical services, and increased concern for infection. Additionally, affordable family housing options are limited, and families who need to relocate close to the hospital for the 28-day cycle face a significant financial burden. Therefore, we recommended that the full 28-day cycle be administered inpatient in order to address these safety concerns and to build staff experience with blinatumomab infusions. As part of the initial barrier assessment, the research team sought to understand LMIC institutional funding mechanisms to ensure equal access for patients outside the private sector and identify charitable opportunities to prevent additional costs from being an insurmountable barrier to medication delivery. This approach was agreed upon with pilot LMIC centers before program initiation and will be an important factor to follow over time and determine the impact on implementation success.

The goal of this implementation study is to develop an in-depth understanding of key determinants and strategies contributing to the successful implementation of blinatumomab in LMICs. Analyses and subsequent iterative adaptation will demonstrate the value of a diverse stakeholder approach and help to understand the mechanism and processes essential for the effective translation of novel therapies. The resulting “Implementation Roadmap” is a critical framework to facilitate safe and effective scale-out of these types of programs to a wider range of resource-constrained settings. Although this initial version of the model is specific to blinatumomab, by defining the core elements related to complex therapy administration, ultimately this model can also be used to help translate other complex novel therapies in pediatric cancer and other disease processes, such as benign hematologic or neurologic diseases, in a resource-limited setting. In terms of impact, this inclusive approach will not only achieve the goal of improving drug provision, but it will facilitate achievable adoption, advance the workforce skillset for management of novel therapies, and promote long-term sustainability that will ultimately improve pediatric cancer survival through increased cure and decreased treatment toxicity associated with traditional therapies.

## Conclusions

**Fig. 2:**
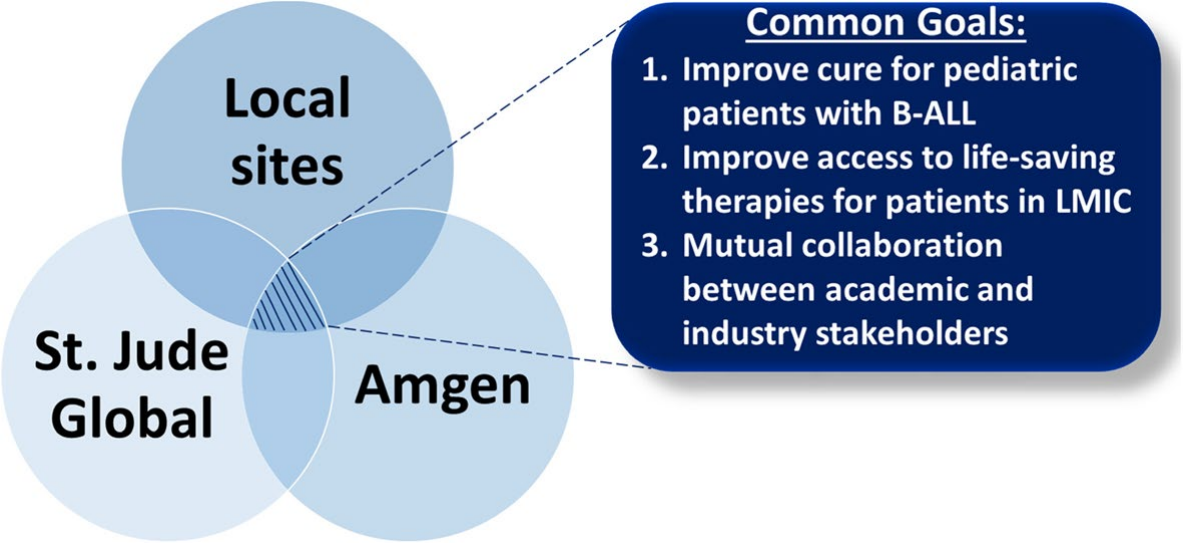
Stakeholder alignment, common goals, and value added by the BHAP program and INOVATING effort

While the last three decades have seen numerous innovative therapies making their way into the pediatric oncology space, the impact of modern-day research is significantly delayed for patients living in LMICs. As the vision for pediatric cancer continues to evolve on a global scale, through efforts such as the World Health Organization (WHO) Global Initiative for Childhood Cancer [37] and the Global Platform for Access to Childhood Cancer Medicines [38], it is imperative that proposed approaches embody the scientific rigor and reproducibility needed to not only meet the growing demands of evidence-based scalability but also engage strategic capacity building within the existing global network. This innovative study uses mixed methods to identify the crucial elements of novel therapy translation to resource-limited settings and is a key example of how major interdisciplinary, academic, and industry partnerships play a crucial role in bridging this difficult gap. Although there is a unique value added for each participating collaborator group, this team has demonstrated the potential impact of collaborator alignment on the common goal of facilitating the translation of evidence-based interventions that improve access and survival for pediatric cancer patients on a global level (Fig. 2).

## Data Availability

Not applicable.

## Abbreviations

ALL: Acute lymphoblastic leukemia
BHAP: Blincyto® Humanitarian Access Program
CFIR: Consolidated Framework for Implementation Research
ERIC: Expert Recommendations for Implementing Change
CITI: Collaborative Institutional Training Initiative
